# The impact of the Hsp67Bc gene product on Drosophila melanogaster longevity, fecundity, and acute heat stress tolerance

**DOI:** 10.18699/VJGB-22-21

**Published:** 2022-03

**Authors:** D. Malkeyeva, S.A. Fedorova, E. Kiseleva

**Affiliations:** Institute of Cytology and Genetics of the Siberian Branch of the Russian Academy of Sciences, Novosibirsk, Russia; Institute of Cytology and Genetics of the Siberian Branch of the Russian Academy of Sciences, Novosibirsk, Russia; Institute of Cytology and Genetics of the Siberian Branch of the Russian Academy of Sciences, Novosibirsk, Russia

**Keywords:** Drosophila longevity, thermal stress tolerance, elevated temperature, heat stress, small heat shock proteins, autophagy, продолжительность жизни Drosophila, устойчивость к температурному стрессу, повышенная температура, тепловой стресс, малые белки теплового шока, аутофагия

## Abstract

Drosophila melanogaster Hsp67Bc is a heat- and cold-inducible small heat shock protein that participates in the prevention of aggregation of misfolded proteins and in macroautophagy regulation. Overexpression of the Hsp67Bc gene has been shown to enhance macroautophagy in Drosophila S2 cells, and the deletion of this gene leads to the formation of a slightly increased number of autophagic vacuoles in the fruit f ly brain neurons. Recently, we found that Hsp67Bc-null D. melanogaster f lies have poor tolerance to cold stress (0 °C) of various durations. In the present work, we investigated how the Hsp67Bc gene deletion affects the f itness of fruit f lies under normal conditions and their tolerance to elevated temperatures at different developmental stages. Larvae and pupae were not adversely affected by the Hsp67Bc gene deletion, and adult Hsp67Bc-null f lies showed an extended lifespan in comparison with the control at normal (24–25 °C) and elevated temperature (29 °C), and after acute heat stress (37 °C, 2 h). At the same time, the fecundity of the mutant females was lower by 6–13 % in all tested environments, except for permanent maintenance at 29 °C, where the mean numbers of eggs laid by the mutant and control f lies were equal. We explain this phenomenon by a reduced number of ovarioles in Hsp67Bc-null females and enhanced macroautophagy in their germaria, which promotes the death of forming egg chambers. In addition, short heat stress (37 °C, 2 h), which increased the control line’s longevity (an effect common for a wide range of organisms), had a negative impact on the lifespan of Hsp67Bc-null f lies. Therefore, Hsp67Bc-null D. melanogaster have an extended lifespan under normal and elevated temperature conditions, and reduced fecundity and thermal stress tolerance.

## Abstract (ru)

Hsp67Bc Drosophila melanogaster – индуцируемый в ответ на тепловой и холодовой стресс малый белок теплового шока, участвующий в предотвращении агрегации поврежденных белков и в регуляции макроаутофагии. Было показано, что повышенная экспрессия гена Hsp67Bc стимулирует макроаутофагию в клетках S2 дрозофилы, а его делеция приводит к небольшому увеличению количества аутофагических вакуолей в нейронах мозга мух. Недавно нами обнаружено, что нуль-аллельные по гену Hsp67Bc особи D. melanogaster имеют сниженную устойчивость к холодовому стрессу (0 °C) различной длительности. В настоящей работе мы исследовали, как наличие делеции в гене Hsp67Bc повлияет на жизнеспособность D. melanogaster в нормальных условиях и на их устойчивость к повышенной температуре на разных стадиях развития. Делеция Hsp67Bc не оказала на личинок и куколок дрозофил неблагоприятного воздействия; нуль-аллельные по гену Hsp67Bc имаго имели увеличенную по сравнению с контролем продолжительность жизни при нормальной (24–25 °C) и повышенной (29 °C) температуре, а также после кратковременного теплового стресса (37 °C, 2 ч). В то же время плодовитость мутантных самок была снижена на 6–13 % по сравнению с контролем при всех исследованных температурных режимах, за исключением постоянного содержания при 29 °C, при котором среднее число откладываемых яиц не различалось между контрольной и мутантной линиями. Мы связываем этот феномен со сниженным количеством овариол у нуль-аллельных по гену Hsp67Bc самок, а также с усиленной макроаутофагией в их гермариях, приводящей к росту числа гибнущих формирующихся яйцевых камер. Кроме того, кратковременный тепловой стресс (37 °C, 2 ч), приводивший к увеличению продолжительности жизни D. melanogaster контрольной линии (что является распространенной реакцией у живых организмов), отрицательно влиял на продолжительность жизни мух с делецией Hsp67Bc. Таким образом, D. melanogaster с делецией в гене Hsp67Bc имеют увеличенную продолжительность жизни в нормальных условиях и при повы- шенной температуре и сниженные плодовитость и устойчивость к температурному стрессу.

## Introduction

During ontogenesis, all living organisms experience stress. The effects of stress-inducing agents on cells include oxidative modification of proteins, which leads to their misfolding (Jolly, Morimoto, 2000). Misfolded proteins are detrimental to the cell because they may gain deleterious biological functions and are prone to forming insoluble aggregates (Jolly, Morimoto, 2000). To maintain homeostasis, cells synthesize heat shock proteins (HSPs): a group of conservative proteins that ensure correct folding of peptides, prevent aggregation of denatured proteins, and resolubilize protein aggregates (Jolly, Morimoto, 2000). This response is universal among all the known proand eukaryotes (Lindquist, 1986). Expression of the majority of HSP genes is up-regulated in stressful conditions such as heat and cold stress, hypoxia, bacterial and viral infections, and oxidative stress (Lindquist, 1986; Sørensen et al., 2003). Some HSPs are constitutively expressed and are necessary for growth and development of organisms under normal conditions (Kampinga et al., 2009; Sarkar et al., 2011). In a study by Raut et al. (2017) on Drosophila, knockdown of 42 out of 95 tested HSP genes led to F1 lethality indicating their crucial role in the fly development.

Drosophila melanogaster Hsp67Bc belongs to the small heat shock protein family of HSPs (Vos et al., 2016). It shares a function of preventing damaged protein aggregation with other members of the family (Vos et al., 2016). In addition, Hsp67Bc was shown to be involved in the regulation of macroautophagy – a conservative catabolic process allowing the recycling of cytoplasm components – alongside Starvin protein (Carra et al., 2010; Parzych, Klionsky, 2014). Overexpression of the Hsp67Bc gene separately or together with stv resulted in protein synthesis inhibition and macroautophagy stimulation (Carra et al., 2010).

Our studies on the Hsp67Bc gene deletion in D. melanogaster revealed that in brain neurons of Hsp67Bc-null flies infected by a pathogenic Wolbachia bacteria strain wMelPop, the number of autophagosomes and autolysosomes (organelles formed in the process of macroautophagy that sequester cytoplasm components and digest them) was increased, and the cross-sectional area of autolysosomes was more than 1.5-fold larger than in the control line with the wild-type Hsp67Bc gene (Malkeyeva et al., 2021). These observations may indicate that in the absence of the Hsp67Bc gene product, macroautophagy is slightly enhanced and autophagosome maturation process is affected. Furthermore, we showed that the Hsp67Bc gene product plays an important role in tolerance to cold stress in the fruit fly (Malkeyeva et al., 2020). Hsp67Bc-null adult flies needed more time to recover from chill coma than the control flies, and adult females with the Hsp67Bc gene deletion had a 1.6–3-fold lower survival after cold stress of various durations (2, 4, and 12 h at 0 °C) as compared to the control line (Malkeyeva et al., 2020).

In this study, we investigated fitness of Hsp67Bc-null D. melanogaster under normal conditions (24–25 °C) and their tolerance to elevated temperatures (29 °C or 2 h at 37 °C) at different stages of ontogenesis (larva, pupa, and imago). We found that the adult mutant flies had an increased lifespan at all the tested temperatures, in comparison with the control line with an intact Hsp67Bc gene. Hsp67Bc-null adult flies, however, had slightly reduced fecundity under normal conditions and after heat stress (37 °C, 2 h) and were negatively affected by acute heat stress (37 °C, 2 h) that prolonged longevity of the control line. Thus, despite having extended lifespan in comparison with the control line under all tested conditions, Hsp67Bc-null flies had lower fecundity and were less tolerant to acute heat stress

## Materials and methods

Drosophila melanogaster lines. In this study, we used Hsp67Bc-null D. melanogaster line Hsp67Bc-0 we created by an imprecise excision of a P-element located in proximity to the Hsp67Bc gene transcription start. Fly line Hsp67Bc-2 containing a wild-type variant of Hsp67Bc obtained by a precise cutting out of the mentioned P-element was used as a control. The procedure for obtaining the fly lines is described in our recent article (Malkeyeva et al., 2020).

Heat stress applied to larvae and pupae. For these experiments, wandering late 3rd instar (L3) larvae were transferred from their rearing vials to the walls of vials with fresh cornmeal-agar medium, at 20 per vial. The larvae were then either directly transferred to a 37 °C environment (water bath in an incubator) for 2 h incubation or allowed to first reach the developmental stage that was to be treated. In particular, these were pupal stages P1–P2 (white prepupae, 1–2 h after pupation), P5 (18–20 h after pupation), or P7–P8 (46–48 h after pupation) (Bainbridge, Bownes, 1981). The cottons sealing the vials were slightly moisturized with water before the start of heat treatment to prevent drying of the larvae and pupae. After the heat stress treatment, the flies were kept at 24–25 °C until eclosion. Survivors to the pupa stage (in case of late L3 treatment) and to the adult stage (for all treatment groups) were then counted. In each experiment, 39–107 flies of each genotype were used.

Analysis of the lifespan and fecundity of adult D. melanogaster kept at either normal or elevated temperature. The flies were collected from rearing vials on the 1st day after eclosion and placed into vials with a fresh cornmeal-agar medium, at eight males and eight females per vial. The flies then underwent one of four treatments. The 1st group was kept at 24–25 °C (normal conditions); the 2nd group was subjected to heat treatment at 37 °C for 2 h at 1 day of age and then was returned to the 24–25 °C environment; the 3rd group was heat treated (37 °C, 2 h) at 7 days of age, then returned to the 24–25 °C environment; the 4th group was transferred to a 29 °C environment at 1 day of age and kept at the elevated temperature. Each experimental group contained 45–62 males and 51–62 females of relevant genotypes.

All the flies were kept under the specified conditions until the death of all individuals, with survivors transferred to fresh food daily or every other day. In parallel, fecundity was measured in these Drosophila starting from day 2 in the 1st, 2nd, and 4th groups and starting from day 8 in the 3rd group (one day after the heat treatment). D. melanogaster females were allowed to lay eggs for 24 h in vials with fresh medium, then the parents were transferred to new food, and the eggs were counted. The number of eggs in a vial was then divided by the number of females that oviposited in that very vial. The egg per female ratio was evaluated on days 2–11, 15–17, and 22–24 in the 1st, 2nd, and 4th experimental groups; in the 3rd group, the ratio was measured on days 8–10, 13–15, and 20–22.

Protein starvation assay. For this assay, newly eclosed adult D. melanogaster individuals were collected every 2 h from their rearing vials and transferred to vials with proteinfree medium containing 100 g/L sucrose, 5 g/L agar and 0.78 g/L methyl 4-hydroxybenzoate. The flies were transferred to a fresh medium every other day.

LysoTracker Red (LTR) staining. On the 5th and 15th days of the protein starvation experiment, ovaries of the starved adult D. melanogaster females and females kept on standard food were dissected in 0.01 M PBS (Medigen) (pH 7.4) and stained with 100 nM LysoTracker Red DND- 99 (Life Technologies) and DAPI. The LTR staining was performed as follows: the dissected ovaries were first placed into a droplet of a 100 nM LTR solution in 0.01 M PBS for 10 min incubation, washed thrice in PBS, and then fixed in 4 % paraformaldehyde for 20 min; next, the ovaries were washed three times with a 0.1 % Triton X-100 solution in PBS and mounted on a slide with a drop of DAPI-containing SlowFade Gold Antifade Mountant (Thermo Fisher Scientific). To make sure the antifade mountant penetrates inner ovarioles, we let the ovaries stay without a cover slip for ~15 min before sealing them under it with nail polish. The samples were stored in the dark at 4 °С until analysis under the LSM 780 confocal microscope (Zeiss) with the Plan-Apochromat 20x/0.8 M27 objective.

Statistical analyses. Survival and recovery curves were compared by the log-rank test. The fecundity, lifespan, number of ovarioles, and number of dying egg chambers per ovariole datasets were tested for normality by the Shapiro–Wilk test; normally distributed data were compared by the heteroscedastic t test; data with non-normal distribution were compared by the Mann–Whitney U test. Differences in fecundity between the control and mutant fly lines throughout the experiment were evaluated at each point by the heteroscedastic t test, followed by the Benjamini–Krieger–Yekutieli method to control the false discovery rate. Analyses of the proportion (%) of LTR-positive germaria obtained in the LTR-staining experiments were performed by the chi-squared test. Differences were considered statistically significant at p ≤ 0.05.

The Shapiro–Wilk test and the Mann–Whitney U test were conducted using Statistics Kingdom statistics calculators (https://www.statskingdom.com).

## Results

**Fig. 1.**
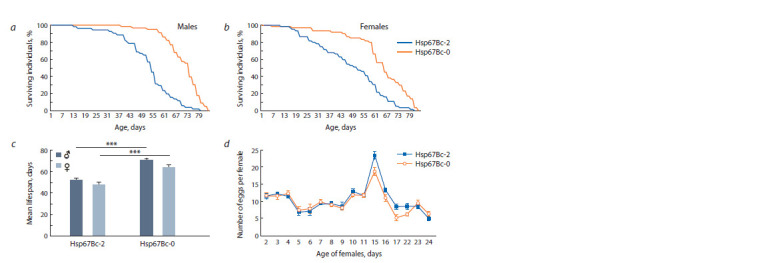
The survival, lifespan, and fecundity of Hsp67Bc-null (Hsp67Bc-0) and control (Hsp67Bc-2) D. melanogaster under normal conditions (24–25 °C). Survival curves of males (a) and females (b); the mean lifespan (c); fecundity (eggs per female) dynamics of mutant and control females throughout the first month of life (d ). The error bars denote standard error of the mean (SEM). *** p ≤ 0.001.

Hsp67Bc-null D. melanogaster under normal conditions To expand our knowledge on the functions of Hsp67Bc in the fruit fly we created a D. melanogaster line carrying a deletion of almost the entire Hsp67Bc gene (described in detail in our recent article (Malkeyeva et al., 2020)). The flies carrying the deletion in the Hsp67Bc gene in the homozygous state (Hsp67Bc-0) were viable and fertile, and had no visible morphological deviations from the control. The Hsp67Bc-0 line had extended longevity under normal conditions (24–25 °C) as compared to the control Hsp67Bc-2 line (Fig. 1, a–c). The mean lifespan of Hsp67Bc-null D. melanogaster significantly exceeded that of the control by 35 % in males and by 34 % in females at 24–25 °C (see Fig. 1, c). Thus, it was 70.9 ± 1.3 days in the mutant males as compared to 52.4 ± 1.9 days in the control males ( p < 0.001) and 63.9 ± 2.2 days in Hsp67Bc-0 females, compared to 47.8 ± 2.4 days in the control line ( p <0.001). On the contrary, the mean fecundity measured during the first month of life was 5.9 % lower ( p = 0.809) in Hsp67Bc-0 females than in the control (see Fig. 1, d ).

Because under normal conditions the absence of the Hsp67Bc gene increased the mean lifespan of the flies while causing only a minor decrease in fecundity, and because no cases of the loss of this gene in wild fruit fly populations have been reported to date, a question arose about the role of the Hsp67Bc gene in D. melanogaster. It is known that heat shock proteins (which include Hsp67Bc) are essential for stress tolerance in all the living organisms (Lindquist, 1986; Sørensen et al., 2003). In our previous study, we discovered involvement of the Hsp67Bc gene product in cold stress tolerance in D. melanogaster (Malkeyeva et al., 2020). In addition, the Hsp67Bc gene expression was shown to increase in response to heat stress (Vos et al., 2016). Therefore, we decided to investigate the impact of the deletion in the Hsp67Bc gene on elevated temperature tolerance in the flies.

The effect of heat stress on survival of Hsp67Bc-null larvae and pupae

According to FlyBase (https://flybase.org), the Hsp67Bc gene expression levels are the highest in wandering 3rd instar (late L3) larvae and pupae of D. melanogaster, in particular, white prepupae, 12 h pupae, and 48 h pupae. We decided to check how heat stress would affect Hsp67Bc-0 flies at those stages of development, in addition to the adult stage.

**Fig. 2.**
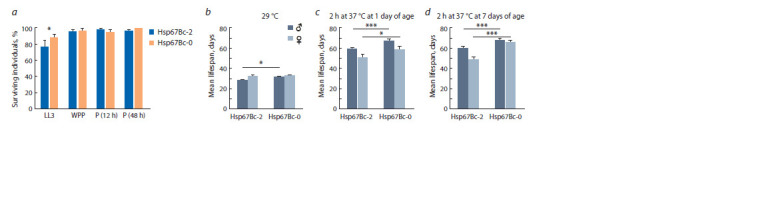
Survival rates of larvae, white prepupae, and pupae of Hsp67Bc-null (Hsp67Bc-0) and control (Hsp67Bc-2) f lies after acute heat stress (37 °C, 2 h), and the mean lifespan of adult mutant and control f lies under different regimens involving heat treatment a, the proportion (%) of wandering L3 larvae (LL3), white prepupae (WPP), 11–13 h pupae (P (12 h)), and of 47–49 h pupae (P (48 h)) surviving to the adult stage after 2 h of heat treatment (37 °C); b, the mean lifespan of the adult males and females constantly kept at 29 °C; c, the mean lifespan of the adult f lies kept at 24–25 °C after 2 h heat treatment (37 °C) at 1 day of age; d, the mean lifespan of the adult f lies kept at 24–25 °C after 2 h heat treatment (37 °C) at 7 days of age. The error bars represent SEM. * 0.010 < p ≤ 0.050; *** p ≤ 0.001.

The larvae and pupae were placed in a 37 °C environment for 2 h, after which they were returned to 24–25 °C to recover and continue development. The survival rates of the larvae and pupae were computed as a proportion (%) of eclosed individuals (Fig. 2, a). The mean survival rates to adult stage were similar between the control and Hsp67Bc-null pupae, varying between 95.0 ± 2.9 % (12 h Hsp67Bc-0 pupae) and 100 % (48 h Hsp67Bc-0 pupae). Statistically significant differences were observed between the survival rates of the control and mutant flies at wandering L3 larva stage: the mutant larvae showed higher survival rate as compared to the control line (88.9 ± 3.1 % in the Hsp67Bc-0 line against 76.6 ± 8.3 % in the control, p = 0.044).

The impact of elevated temperature on Hsp67Bc-null adult flies

and pupae, Hsp67Bc-0 and control adult flies were subjected to one of the two variants of elevated temperature treatment. The first variant was life-long maintenance at 29 °C starting from one day of age; the second variant included heat stress (2 h at 37 °C) at either one or seven days of age with subsequent maintenance at 24–25 °C until death of all individuals. The fly ages for heat stress treatment (2 h at 37 °C) were chosen based on FlyBase (https://flybase.org) data indicating that the Hsp67Bc protein levels are much higher in 1-day-old flies than in 7-day-old flies

**Fig. 3.**
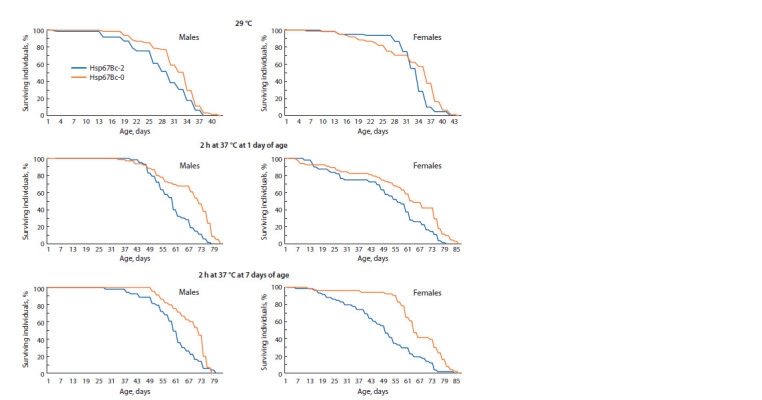
Survival curves of adult Hsp67Bc-null (Hsp67Bc-0) and control (Hsp67Bc-2) f lies kept at 29 °C or at 24–25 °C after 2 h heat treatment (37 °C) at either 1 or 7 days of age.

Constant maintenance at 29 °C significantly shortened the lifespan of both control and Hsp67Bc-null flies, as compared to maintenance under normal conditions (24–25 °C) without heat treatment (see Fig. 2, b, Fig. 1, c). The mean lifespan of males was 28.1 ± 0.9 days in the control line and 31.3 ± 0.8 days in the mutant line. Still, the mean lifespan of Hsp67Bc-null males was 11.5 % higher than that of the control line at 29 °C ( p = 0.010), and the mutant males passed 50 % survival between days 33 and 34 of the experiment, whereas the control ones had passed it already between days 29 and 30 (Fig. 3). Females of the control line had a mean lifespan of 32.4 ± 0.7 days and Hsp67Bc-0 females had a mean lifespan of 32.5 ± 1.1 days. Unlike males, females of the control and mutant lines had similar survival dynamics and lifespan at 29 °C (see Fig. 2, b, Fig. 3). Of note, although the mean lifespan of Hsp67Bc-null Drosophila was exceeding or equal to that of the control flies at the 29 °C environment, the reduction of longevity caused by maintenance at the elevated temperature (29 °C) was more prominent in the mutant flies than in the control ones. Thus, maintenance at 29 °C reduced the lifespan of Hsp67Bc-2 males and females 1.9-fold and 1.5-fold, respectively, as compared to normal conditions (24–25 °C) without heat stress, whereas the decline was 2.3-fold in Hsp67Bc-null males and 2.0-fold in Hsp67Bc-0 females (see Fig. 1, c, Fig. 2, b).

Heat shock (37 °C, 2 h) did not cause death in neither the control nor Hsp67Bc-null fly line. The mean lifespan of Hsp67Bc-0 flies was higher as compared to the control among both males and females at both variants of heat treatment (at one or seven days of age) (see Fig. 2, c, d ). Survival dynamics also significantly differed between the lines with p ≤ 0.010 (see Fig. 3). The mean lifespan of Hsp67Bc-0 males heat-treated at one or seven days of age was higher than that of the control males by ~13 % ( p < 0.001) and was 67.0 ± 1.7 days in Hsp67Bc-0 males heat-shocked at one day of age and 67.9 ± 1.3 days in the mutant males heat-shocked at seven days of age (see Fig. 2, c, d ). The mean lifespan of Hsp67Bc-null females that underwent heat stress at one day of age exceeded that of the control females by ~16 % (58.9 ± 2.8 days, p = 0.014); between the control and mutant females heat-treated at seven days of age, the difference in the mean lifespan was ~35 % (65.4 ± 2.0 days, whereas that of Hsp67Bc-2 females was 48.5 ± 2.4 days, p <0.001) (see Fig. 2, c, d ). Of note, the applied heat stress (37 °C, 2 h) had a different impact on the control and Hsp67Bc-null flies. In comparison with the maintenance under normal conditions (24–25 °C, without treatment), it increased longevity of the control males and females by 1.5–14.5 % (see Fig. 1, c, Fig. 2, c, d ). On the contrary, in the Hsp67Bc-0 line, heat stress at 37 °C reduced the mean lifespan of females treated at one day of age by 7.8 %, and the mean lifespan of males heat-shocked at one and seven days of age by 5.5 and 4.2 %, respectively (see Fig. 1, c, Fig. 2, c, d ).

These findings may suggest that even though the Hsp67Bc gene deletion causes an increase in the lifespan of flies at both normal and elevated temperature, it has a detrimental effect on tolerance to acute heat stress, which normally improves the longevity of flies (Hercus et al., 2003; Le Bourg, 2011; Sarup et al., 2014).

The effect of elevated temperature on D. melanogaster fecundity

In parallel with lifespan and survival, we measured fecundity of the control and Hsp67Bc-null females as the number of eggs laid in each vial within 24 h divided by the number of females kept in those very vials. The mean egg per female ratio calculated throughout the experiment did not statistically differ between the control and mutant lines in any of the heat treatment groups (29 °C, 2 h at 37 °C at one day of age, and 2 h at 37 °C at seven days of age). Nevertheless, Hsp67Bc-null females had slightly reduced fecundity as compared to the control flies after being subjected to heat shock (37 °C, 2 h). The differences between the lines were more prominent than at 24–25 °C without treatment. Thus, the mean number of eggs per female was 10.5 % lower in the mutant flies that underwent the heat treatment at one day of age as compared to the control (8.71 eggs/female in Hsp67Bc-0 line and 9.73 eggs/female in Hsp67Bc-2 line, p = 0.564); in the mutant flies subjected to heat stress at seven days of age, this value was 12.8 % (9.77 eggs/female in Hsp67Bc-0 line and 11.2 eggs/female in the control, p = 0.427).

**Fig. 4.**
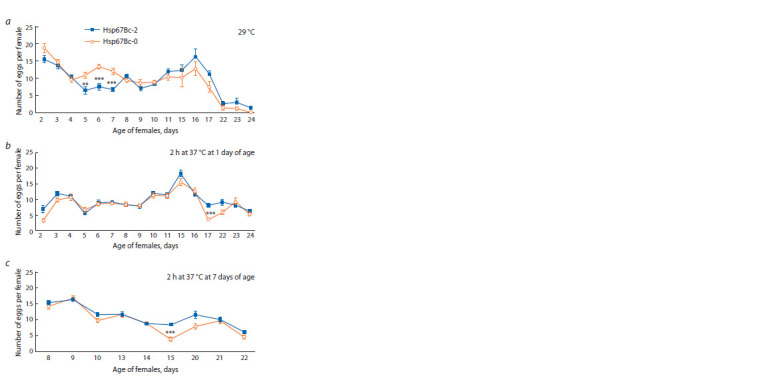
Fecundity (eggs per female) measured throughout the f irst month of life of the Hsp67Bc-null (Hsp67Bc-0) and control (Hsp67Bc-2) females kept at 29 °C (a) or at 24–25 °C after 2 h heat treatment (37 °C) at either 1 day (b) or 7 days (c) of age. The error bars denote SEM. ** 0.001 < p ≤ 0.010; *** p ≤ 0.001.

The egg/female ratio measured each day significantly differed between the mutant and control lines only on some of the days of the experiment (Fig. 4). It is worth mentioning that heat shock (37 °C, 2 h) at one day of age was detrimental for the fecundity of females. The next day after the heat treatment, the number of laid eggs per female was much less than under normal conditions without treatment in both the control and mutant lines (see Fig. 4, b, Fig. 1, d ). The decrease was more prominent in Hsp67Bc-null flies (72 % decrease in Hsp67Bc-0 line as compared to 40 % reduction in the control line).

In search for the cause of the reduced mean fecundity in Hsp67Bc-null females kept at 24–25 °C, we analyzed the morphology of ovaries in the control and mutant lines. In the ovaries of both the control and mutant flies, egg chambers at all stages of oogenesis were present. However, the mutant females had lower number of ovarioles than the control ones. Fiveand 15-day-old Hsp67Bc-2 females had 16.9–18.4 ovarioles per ovary, whereas Hsp67Bc-0 females had 14.6–16.2 ovarioles per ovary ( p = 0.680 in case of the 5-day-old flies and p < 0.001 in case of the 15-day-old flies). This finding may partially explain the difference in the fecundity of the two lines

The number of ovarioles may be influenced by nutrient deprivation in D. melanogaster (Sarikaya et al., 2012). Dietary restriction stimulates macroautophagy, a process of intracellular component degradation, in regulation of which Hsp67Bc was shown to participate (Amano et al., 2006; Carra et al., 2010; Kroemer et al., 2010). Our recent studies on macroautophagy revealed a slight increase in autophagic vacuole number in the brain of adult Hsp67Bc-null flies (Malkeyeva et al., 2021). Therefore, we next decided to study the morphology of the control and Hsp67Bc-null D. melanogaster ovaries under the stress of protein starvation.

The impact of Hsp67Bc gene deletion on starvation-induced macroautophagy in D. melanogaster ovaries

**Fig. 5.**
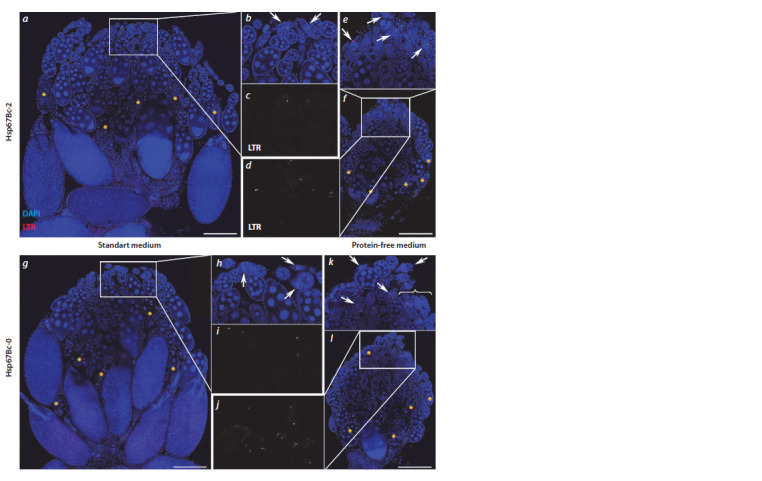
LTR-labelled ovaries of Hsp67Bc-null (Hsp67Bc-0) and control (Hsp67Bc-2) adult D. melanogaster females kept on either the standard or protein-free medium for 5 days. a, an ovary of a control female kept on the standard medium; b, c, the magnif ied white rectangle from panel a showing DAPI (blue) and LTR (red) channels (b) and a separate LTR channel (c); d–f, an ovary (f ) of an Hsp67Bc-2 female kept on the protein-free medium, and its magnif ied fragment (white rectangle from panel f ) showing DAPI and LTR channels (e) and a separate LTR channel (d ); g–l, same as a–f, for the Hsp67Bc-0 line. Yellow asterisks denote egg chambers with highly condensed and/or fragmented nuclei; white arrows indicate LTR-positive germaria; because too many LTR-positive germaria are present in panel k, not all of them are indicated by arrows, and three of them are indicated by a brace. Scale bars are 200 μm.

It is known that starvation, including protein deprivation, induces macroautophagy in Drosophila ovaries at two nutrient status checkpoints: germarium and mid-oogenesis (Hou et al., 2008), which then leads to oogenesis slowdown and to an increase in the number of egg chambers eliminated from oogenesis (Barth et al., 2011). The discarded egg chambers degrade through apoptosis with the participation of autophagy (Bolobolova et al., 2020). To evaluate macroautophagy intensity, we used the LysoTracker Red DND-99 (LTR) dye, which had been shown to label acidic organelles, such as lysosomes and autolysosomes, in D. melanogaster (Scott et al., 2004; Klionsky et al., 2007). Massive acidification of the cytoplasm signifies death of forming egg chamber cells. We estimated the percentages of LTR-positive germaria in the control and Hsp67Bc-null flies kept on standard food and after five (Fig. 5) and 15 days of protein starvation

As compared to the control line, in the ovaries of Hsp67Bcnull females kept on either the standard or protein-free medium, a higher number of LTR-positive germaria was observed (Fig. 6, see Fig. 5). During early oogenesis, the Hsp67Bc gene deletion resulted in a 1.2- to 1.5-fold increase in the LTRpositive– germaria proportion (see Fig. 6). Thus, in 5-day-old Hsp67Bc-0 females kept on the standard medium, 32.1 % of germaria were LTR-positive, relative to 21.1 % in the control Hsp67Bc-2 line ( p = 0.066); in 5-day-old starved Hsp67Bcnull female ovaries, the percentage of LTR-positive germaria was as high as 77.9 % but was only 59.3 % in Hsp67Bc-2 females ( p = 0.045). In 15-day-old Hsp67Bc-null females kept on the standard food, LTR-positive germaria constituted 31.1 %, whereas in the control line, this proportion was 24.3 % ( p = 0.348); in 15-day-old starved flies, the percentages of LTR-positive germaria in ovaries were 73.3 % in the Hsp67Bc-0 line and 60.4 % in the Hsp67Bc-2 line ( p = 0.226). These data reflect an increase in macroautophagy intensity in the germaria of Hsp67Bc-null females.

During mid-oogenesis, we noted a decrease in the number of egg chambers with highly condensed chromatin (see Fig. 5), which is a marker of apoptotic cell death, in Hsp67Bc-null flies in comparison with the control, except for the 5-day-old females kept on the standard medium. In 5- and 15-day-old starved and in 15-day-old normally fed Hsp67Bc-null flies, the mean number of apoptotic egg chambers per ovariole was slightly lower than that in the control flies, the difference being significant only in 5-day-old starved flies (Fig. 6). Thus, the observed egg chamber apoptosis was 37 % lower in 5-day-old starved mutant females than in the control females ( p = 0.008). In 15-day-old starved flies, the mean number of apoptotic egg chambers per ovariole was 11 % lower in the Hsp67Bc-0 flies than that in the control individuals ( p = 0.528); in 15-day-old females kept on the standard food, this number was 35 % lower in the mutant flies than in the Hsp67Bc-2 line ( p = 0.131). Although we observed a lower mean number of apoptotically dying mid-oogenesis egg chambers in Hsp67Bc-null flies, we can hypothesize that this phenomenon is related to the observed increased apoptosis of forming egg chambers in germaria during early oogenesis in the mutant flies.

## Discussion

In this study, we investigated the impact of the Hsp67Bc gene deletion on D. melanogaster fitness under normal conditions and on their heat-stress tolerance. Hsp67Bc-null flies showed extended lifespan as compared to the control line under normal conditions (24–25 °C), elevated temperature conditions (29 °C), and after acute heat stress (37 °C, 2 h) (see Fig. 1, Fig. 2). At the same time, the mean fecundity of the mutant females was slightly reduced at 24–25 °C without heat treatment and after the short heat stress (Fig. 1, d, Fig. 4, b, c).

The observed statistically insignificant decrease in Hsp67Bcnull female fecundity can be explained by a combination of the following factors. First, the mutants had reduced number of ovarioles, a trait that was reported by other researchers to result in lower egg yield (Yamamoto et al., 2021). Second, the quantity of LTR-positive germaria was higher in Hsp67Bcnull females as compared to the control line (Fig. 6), which indicates increased macroautophagy and enhanced death of forming egg chambers resulting in less eggs (Drummond- Barbosa, Spradling, 2001; Nezis et al., 2009). Contrary, in the mutant females, a lower number of mid-oogenesis egg chambers dying via apoptosis was present as compared to the control flies (Fig. 6). This last feature of the mutant ovaries may partially compensate the first two in terms of eventual egg yield making the difference between the lines statistically insignificant. In D. melanogaster, ovariole number is determined at the stage of 3rd instar larva and can be influenced by either genetic or environmental factors, such as rearing temperature and diet (Sarikaya et al., 2012).

Nutrition plays an important role in defining the quantity of ovarioles: larvae kept on medium with reduced nutrient level develop into adult flies with less ovarioles (Sarikaya et al., 2012). The decreased ovariole number in Hsp67Bc-null flies reared on the standard food may be caused by impaired larva nutrition due to reduced food intake or uptake, which was not registered in our studies. Alternatively, the number of ovarioles in mutant flies could be affected by slightly increased macroautophagy, which we detected in Hsp67Bc-null fly germaria and, previously, in brain neurons of adult flies with the Hsp67Bc gene deletion (Malkeyeva et al., 2021). It is known that macroautophagy is strongly stimulated in response to starvation (Kroemer et al., 2010); therefore, enhanced macroautophagy on larval stage caused by the absence of the Hsp67Bc gene product may mimic nutrient deprivation conditions leading to formation of less ovarioles. The decrease in apoptotic stage 8 egg chambers in Hsp67Bc-null females may be a result of increased death of forming egg chambers and, hence, enhanced quality control in the germarium resulting in less defective mid-oogenesis egg chambers in Hsp67Bc-null fly ovaries in comparison with the control line.

Extended longevity caused by gene mutations has been reported in D. melanogaster. Lifespan is increased in fruit flies carrying hypomorphic mutations in the InR (insulin-like receptor), chico, and methuselah genes (Lin et al., 1998; Clansy et al., 2001; Tatar et al., 2001). Notably, products of all these genes are involved in macroautophagy modulation via target of rapamycin (TOR) pathway (Clansy et al., 2001; Wang et al., 2015; Graze et al., 2018; Yamamoto et al., 2021), and their down-regulation leads to macroautophagy stimulation. Similarly, macroautophagy stimulation by dietary restriction or TOR kinase inhibition expands lifespan of animals belonging to various taxa (Masoro, 2000; Kapahi et al., 2004). Moreover, it was shown that longevity extension of chico-null D. melanogaster is only possible with intact macroautophagy (Bjedov et al., 2020). In this work, we discovered that the number of LTR-positive germaria was slightly higher in Hsp67Bc-null D. melanogaster ovaries (Fig. 6), which signifies increased macroautophagy. Similarly, our recent study on ultrastructure of neurons in Wolbachia-infected Drosophila brains revealed an increment in the number of autophagic vacuoles in Hsp67Bc-0 fly neurons, which, again, points towards enhanced macroautophagy (Malkeyeva et al., 2021). Bjedov et al. (2020) demonstrated that moderate enhancement of macroautophagy in a complex of tissues increases lifespan in D. melanogaster, while strong and ubiquitous stimulation of macroautophagy shortens it. Hence, the extension of lifespan we observed in Hsp67Bc-null flies may be caused by slight increase in macroautophagy in their tissues.

Although the Hsp67Bc-0 flies had an increased lifespan as compared to the control line under all the tested conditions (normal temperature, elevated temperature, and short heat stress), a decline in longevity was present in the mutant flies that were heat treated in relation to untreated Hsp67Bc-null flies. Heat shock had the opposite effect on the control line, with acute heat stress improving the longevity of the flies. Generally, mild heat (or other stress) treatment of young adults extends Drosophila longevity (Hercus et al., 2003; Le Bourg, 2011; Sarup et al., 2014), as we observed in the control flies. Our experiments with 2–12 h cold treatment of Hsp67Bcnull fly line revealed a decreased cold stress tolerance in the mutants (Malkeyeva et al., 2020). Taken together, our results point towards an adverse impact of the Hsp67Bc gene deletion on short temperature stress tolerance in adult D. melanogaster. While in the laboratory environment flies are rarely exposed to thermal and other stresses, conditions are different in the D. melanogaster natural habitat, where fruit flies may experience a wide variety of extreme stresses including heat shock and chill coma. Therefore, an extended lifespan under normal conditions does not guarantee survival in the wild, as was revealed in a study by Wit et al. (2013). It is important for the survival of poikilothermic animals like Drosophila to be able to cope with thermal stresses. Hence, the loss of the Hsp67Bc gene, the product of which promotes tolerance to acute thermal stresses, though extending lifespan under normal conditions, may be deleterious in a changing environment. Taking into account that D. melanogaster overwinter at the adult stage in temperate regions (Izquierdo, 1991), we assume that the Hsp67Bc gene was not eliminated from the fruit fly genome because of its prominent role in promoting acute heat and cold tolerance in adult flies.

## Conclusion

Here, we studied the effect of the Hsp67Bc gene deletion on D. melanogaster lifespan and fecundity under normal conditions, and their tolerance to elevated temperature and acute heat stress. We did not detect any difference in survival of heat-shocked (37 °C, 2 h) pupae between the mutant and control lines, and the Hsp67Bc-null larvae showed improved survival. Adult Hsp67Bc-null flies had a greater lifespan than the control line at all the tested temperature regimes but lower fecundity and decreased acute heat tolerance. We hypothesize that the lifespan extension is caused by slightly increased macroautophagy in the mutant flies, which we observed in ovaries of Hsp67Bc-deficient Drosophila and – in our earlier work – in the brains of Hsp67Bc-null females. At the same time, the enhanced macroautophagy in germaria, combined with a reduced number of ovarioles, may be the cause of the fecundity reduction in the mutant flies. In conclusion, although the Hsp67Bc gene deletion causes the increase in D. melanogaster lifespan in a stress-free environment, it has a negative effect on fruit fly acute heat stress tolerance, which may negate the longevity benefits in nature habitat, where stresses like extremely high and low temperature are common.

## Conflict of interest

The authors declare no conflict of interest.
